# Supplementary material for: Central inhibition of stearoyl-CoA desaturase has minimal effects on the peripheral metabolic symptoms of the 3xTg Alzheimer’s disease mouse model
Source: Sci Rep. 2024 Apr 2;14:7742. doi: 10.1038/s41598-024-58272-8 (PMC10987571; doi:10.1038/s41598-024-58272-8)
Supplement: Supplementary file 1 — Supplementary Information 1. [file 41598_2024_58272_MOESM1_ESM.docx]

**Supplemental Material**

**Supplemental Figure legends**

**Figure S1: Additional peripheral measurements in 3-month-old WT and 3xTg-AD mice**

**a**-**c** Plasma concentrations of **a** free fatty acids (n=4-5, p=0.9386), **b** triglycerides (n=4-5, p=0.6880), **c** leptin (n=9-11, p=0.0301) between 3-month-old WT and 3xTg-AD mice. **d** the pancreas showed no significant difference. Unpaired t-tests.

Error bars represent mean ± standard error of the mean (SEM). Significance level was set at *p* ≤ 0.05.*p≤0.05.

**Figure S2: Peripheral organ weight in ICV SCDi-treated WT and 3xTg-AD mice**

**a**-**c** Organ weight in grams (g), n=8 animals/group. **a** liver weight showed no significant treatment or strain changes. **b** spleen weight showed significant strain effect (F(1,29) =8,929, p<0.0001). Post hoc analysis showed a significant difference between vehicle WT-V/3xTg-V (p=0.0157) and SCDi treated WT-S/3xTg-S (p=0.0009) but no significant drug effect, 3xTg-V/3xTg-S (p=0.5725), 2-way ANOVA Tukey’s post hoc, **c** the pancreas showed no significant differences between any of the groups.

Error bars represent mean ± standard error of the mean (SEM). Significance level was set at *p* ≤ 0.05.**p≤0.01,****p≤0.0001.
