# Supplementary figures and images for: Central inhibition of stearoyl-CoA desaturase has minimal effects on the peripheral metabolic symptoms of the 3xTg Alzheimer’s disease mouse model
Source: Sci Rep. 2024 Apr 2;14:7742. doi: 10.1038/s41598-024-58272-8 (PMC10987571; doi:10.1038/s41598-024-58272-8)

Supplemental Figure 1

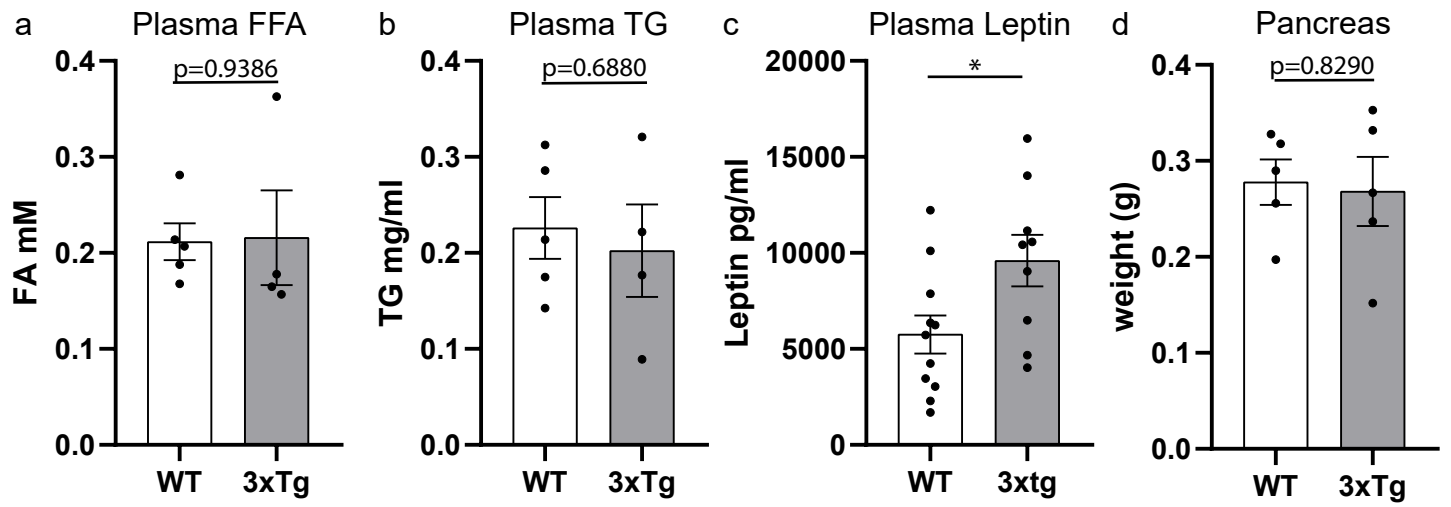

Supplement: Supplementary file 2 — Supplementary Information 2. [file 41598_2024_58272_MOESM2_ESM.pdf]

Supplemental Figure 2

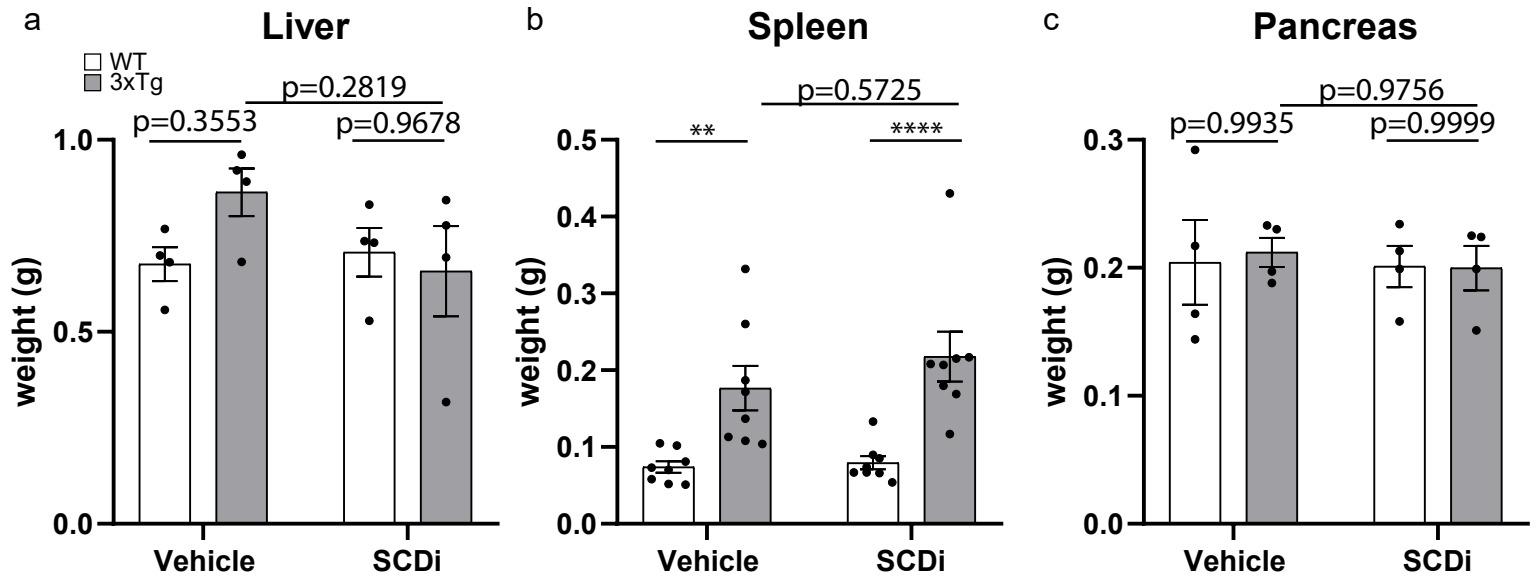

Supplement: Supplementary file 3 — Supplementary Information 3. [file 41598_2024_58272_MOESM3_ESM.pdf]
